# Indications and outcomes of enterovesical and colovesical fistulas: systematic review of the literature and meta-analysis of prevalence

**DOI:** 10.1186/s12893-021-01272-6

**Published:** 2021-05-27

**Authors:** Stefano Granieri, Francesco Sessa, Alessandro Bonomi, Sissi Paleino, Federica Bruno, Andrea Chierici, Ivano Massimiliano Sciannamea, Alessandro Germini, Riccardo Campi, Michele Talso, Antonio Facciorusso, Gianfranco Deiana, Sergio Serni, Christian Cotsoglou

**Affiliations:** 1 General Surgery Unit, ASST Brianza, Vimercate Hospital, Via Santi Cosma e Damiano, 10, 20871 Vimercate, Italy; 2 Unit of Urological Robotic Surgery and Renal Transplantation, Careggi Hospital, University of Florence, Largo Piero Palagi, 1, 50139 Florence, Italy; 3 Department of Experimental and Clinical Medicine, University of Florence, Piazza di San Marco, 4, 50121 Florence, Italy; 4 University of Milan, Via Festa del Perdono, 7, 20122 Milan, Italy; 5 Urology Unit, ASST Brianza, Vimercate Hospital, Via Santi Cosma e Damiano 10, 20871 Vimercate, Italy; 6 General Surgery Unit, ASST Fatebenefratelli-Sacco, Via Giovanni Battista Grassi, 74, 20157 Milan, Italy; 7 Department of Medical Sciences, Gastroenterology Unit, Ospedali Riuniti di Foggia, Viale Luigi Pinto, 1, 71122 Foggia, Italy

**Keywords:** Colovesical fistula, Enterovesical fistula, Surgical management, Systematic review, Meta-analysis

## Abstract

**Background:**

Entero-colovesical fistula is a rare complication of various benign and malignant diseases. The diagnosis is prominently based on clinical symptoms; imaging studies are necessary not only to confirm the presence of the fistula, but more importantly to demonstrate the extent and the nature of the fistula. There is still a lack of consensus regarding the *if, when and how* to repair the fistula. The aim of the study is to review the different surgical treatment options, focus on surgical indications, and explore cumulative recurrence, morbidity, and mortality rates of entero-vesical and colo-vesical fistula patients.

**Methods:**

A systematic review of the literature was conducted according to PRISMA guidelines. Random effects meta-analyses of proportions were developed to assess primary and secondary endpoints. I^2^ statistic and Cochran’s Q test were computed to assess inter-studies’ heterogeneity.

**Results:**

Twenty-two studies were included in the analysis with a total of 861 patients. Meta-analyses of proportions pointed out 5, 22.2, and 4.9% rates for recurrence, complications, and mortality respectively. A single-stage procedure was performed in 75.5% of the cases, whereas a multi-stage operation in 15.5% of patients. Palliative surgery was performed in 6.2% of the cases. In 2.3% of the cases, the surgical procedure was not specified. Simple and advanced repair of the bladder was performed in 84.3% and 15.6% of the cases respectively.

**Conclusions:**

Although burdened by a non-negligible rate of complications, surgical repair of entero-colovesical fistula leads to excellent results in terms of primary healing. Our review offers opportunities for significant further research in this field. *Level of Evidence* Level III according to ELIS (SR/MA with up to two negative criteria).

**Supplementary Information:**

The online version contains supplementary material available at 10.1186/s12893-021-01272-6.

## Background

Enterovesical and colovesical fistulas (EVF, CVF) are an aberrant, pathological communication between the enteric tract and the bladder. They have different etiologies such as neoplasms of the colon and the bladder, inflammatory diseases, pelvic radiation therapy, traumatic and iatrogenic injuries. Among them, the most common is represented by colonic diverticulitis which accounts for 65–79% of cases, whereas 10–20% of cases are due to cancer, most frequently colonic adenocarcinoma [1–3]. Men seem to be more affected than women with a male/female ratio ranging from 2 to 3:1 [4, 5].

Up to 90% of patients suffering from EVF or CVF experience pneumaturia and/or fecaluria [6–9]. Other frequent symptoms are suprapubic pain, dysuria, urgency, and frequency, abdominal tenderness, abdominal mass, weight loss, or cutaneous manifestations in case of Crohn’s disease (CD).

Computed tomography (CT) with oral or rectal contrast, represents the gold standard for diagnosis [5, 8, 10]. A second phase with IV contrast is recommended. Other useful diagnostic tools are represented by colonoscopy and cystoscopy [8, 11, 12]. Both methods are ideal to investigate hollow viscous mucosa and allow biopsies to confirm the suspicion of a cancer-related origin of EVFs/CVFs.

Another useful diagnostic technique is represented by magnetic resonance (MR) especially when the underlying cause of EVF/CVF is Crohn’s disease. Its high sensitivity and specificity in the diagnosis of complex intrabdominal and perianal fistulas is well established [12, 13]; therefore, in the presence of inflammatory bowel diseases it should be preferred over CT.

The treatment of entero-colovesical fistulas is eminently surgical [11, 14]. Once the eventual abdominal fistula-related sepsis has been resolved with broad spectrum/targeted antibiotic therapy and bladder decompression through a Foley catheter, the primary treatment option is the closure of the fistula. Nonoperative management has been advocated for patients unfit for surgery, with mild symptoms, or those who decline surgical indication [15].

Despite a wide spectrum of open and laparoscopic surgical options, a lack of consensus concerning the timing, modalities, and optimal surgical strategy, based on the etiology of the fistula, still exists.

The present study aims to review the different surgical treatment options and to focus on surgical indications. To the best of our knowledge, this is the first meta-analysis in exploring cumulative recurrence, morbidity, and mortality rates of EVF and CVF patients.

## Materials and methods

### Search strategy

A systematic review of the English-language literature was performed according to PRISMA and AMSTAR guidelines [16, 17]. The Medline, Scopus, Cochrane Library and Web of Sciences databases were screened without time restrictions, up to August 10th, 2020. Articles without free full text available were searched through the digital library of the University of Milan, University of Florence, and through direct contact with authors. Hand-search of bibliographies of included studies and previous reviews on the topic was also performed to include additional relevant studies according to our selection criteria. Two investigators (SG, FS) carried out the literature search independently.

### Primary and secondary endpoints

The main aim of this review is to explore the effect of surgical treatments of colovesical and enterovesical fistulas and to summarize the current surgical indications. The primary outcome is represented by fistula recurrence, which is considered an indirect measure of primary healing of the fistula. The secondary outcome is represented by postoperative morbidity and mortality rates.

### Inclusion criteria

We have included studies reporting surgical management of colo-enterovescical fistulas. Both clinical and radiological confirmation has had to be mentioned to deem the study eligible for the review. Benign aetiologies such as postoperative, iatrogenic, inflammatory (Inflammatory Bowel Disease, diverticulitis), and post-radiation therapy, as well as tumor related colovesical/enterovesical fistulas, have been considered.

A specific population (P), intervention (I), comparator (C), outcome (O), and study design (S) (PICOS) framework was specified to define study eligibility, as recommended [16]. In particular, the following criteria were outlined:

*Population (P)* patients with a clinically and radiologically confirmed diagnosis of colovesical or enterovesical fistula;

*Intervention (I)* surgical management of the fistula;

*Comparison (C)* any other non-surgical treatment (this criterion was not mandatory for inclusion of the studies in this review);

*Outcomes (O)* primary healing of the fistula;

*Study design (S)* randomized-controlled or prospective/retrospective cohort studies and case series with more than 10 patients.

### Exclusion criteria

Studies including only patients managed conservatively or with insufficient reporting of the PICOS criteria were excluded. Similarly, studies including pediatric patients (< 18 years), with a mean/median follow-up (FUP) lower than 3 months, non-English language written, and previously published reviews were not deemed eligible.

### Systematic review process

Mendeley reference software (Mendeley Ltd, London, UK) was used to identify and remove duplicates among identified records. After the exclusion of duplicates, three independent reviewers (SP, FB, AC) screened titles and abstracts. An a priori developed screening form was created to guide study selection. Disagreements were solved by a third party (IS), who supervised the systematic review process. Case reports, book chapters, editorials, conference abstracts, pre-clinical studies, previous reviews, and articles not related to the primary endpoint of this review were excluded.

### Data extraction and assessment of included studies

Data were extracted independently by two authors (SG, AB). The following summary data for the included studies were retrieved: name of the authors, year of publication, type of study, demographic data, etiology and type of fistula, diagnostic methods, surgical approach and management, FUP duration, recurrence, morbidity and mortality rates.

In case of disagreement, a further reviewer (AG) helped resolve the disagreement through discussion.

Two authors (SP, AF) independently assessed the quality of evidence provided by each study using the Oxford Center for Evidence-Based Medicine scoring system [18].

### Statistical analysis

The primary outcome measure was represented by the proportion of patients with EVF/CVF recurrence after surgery. A random-effects model based on generic inverse variance method was built to assess the impact of heterogeneity on results. The presence of outliers was investigated, and their effects sizes excluded.

Heterogeneity between studies was quantified by I^2^ statistic and Cochran’s Q test; cut-off values of 25, 50, and 75% were considered as low, moderate, and high, respectively [19]. Sensitivity analysis was performed using the leave-one-out method and Baujat plot was built to visually inspect studies overly contributing to heterogeneity.

Since our systematic review included studies published some time ago as well as recent pieces of evidence, we wanted to explore the effect of surgical practice improvement over time on fistula recurrence through metaregression analysis.

Funnel plots were developed to explore publication bias and Egger’s test of the intercept was used to quantify funnel plots asymmetry. Duval & Tweedie’s trim-and-fill method was adopted to estimate and adjust for the number and outcomes of missing studies each time Egger’s test demonstrated significant asymmetry. P-curve analysis was performed to confirm the results of the aforementioned publication bias assessment.

Statistical analysis was conducted with R statistical software (The Comprehensive R Archive Network—CRAN, ver. 4.0.0 × 64) [20], using “meta”, “metafor”, and “dmetar” packages [21–24].

## Results

**Fig. 1:**
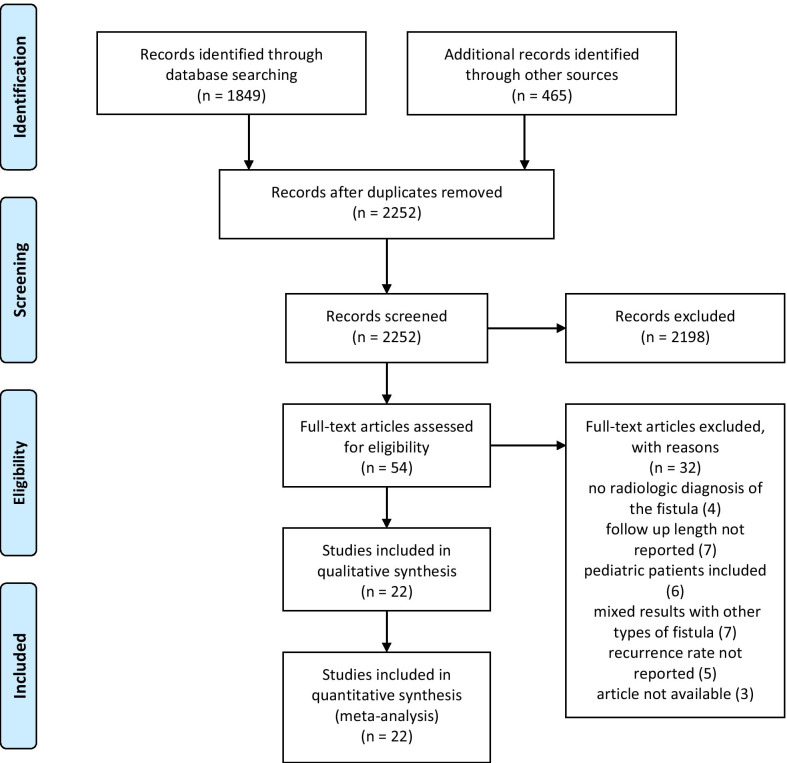
PRISMA flow diagram of selected studies

The PRISMA flow diagram reporting the systematic review process is shown in Fig. 1.

Overall, 2314 articles were preliminarily identified by the literature search. Afer the exclusion of duplicates, titles and abstracts of 2252 records were screened. Fifty four articles were assessed for eligibility. Finally, 22 studies were included in qualitative and quantitative synthesis [2, 9–12, 25–41]. All of them were retrospective. Most of the excluded studies were deemed not eligible due to insufficient reporting of follow-up data, inclusion of pediatric patients, and mixed results with other types of enteric fistulas. Adjusted observational studies were not available, however the cohorts of the included studies were quite similar for principal confounders (age, underlying pathology, comorbidities).

In total, 861 patients undergoing surgery for EVF and/or CVF were included in the study. The male/female ratio was 4:1 and the mean age was 58.7 years. Seven studies reported mean FUP time with an average of 53.6 months; one study reported a FUP range of 2–108 months; finally, the median FUP of the remaining studies was 34 months. Table 1 summarizes patients’ characteristics.

**Table 1:** Studies and patients characteristics

Author	Year of publication	Years of enrollment	Quality of evidence (OCEBM)	Country	No. of surgically treated patients	Mean age	Male:female	Etiology	Type of fistula	
Pollard	1987	1971–1986	4	UK	61	61	36:25	DD 71%; CRC 12.1%; BC 1.5%; CD 6%; PR 4.5%; Appendicitis 3%; Trauma 1.5%	CVF 100%	
McNamara	1990	1952–1986	2b	Canada	61	34.4	38:23	CD 100%	EVF 85.2%; RVF 4.9%; CVF 9.8%	
Holmes	1992	1988–1991	4	UK	11	69	8:3	CRC 63.6%; BC 36.4%	CVF 100%	
McBeath	1994	1980–1991	2b	USA	76	62.1	51:25	DD 59%; Malignancy 9%; PR 7%; Radioneoplastic 8%; CD 9%; Appendicitis 3%; other 6%		
Munoz	1998	1980–1995	4	USA	33	62	56:6	Benign disease 27%; Malignancy 73%		
Vasilevsky	1998	1975–1995	4	Canada	23		8:15	DD 100%	CVF 100%	
Yamamoto	2000	1970–1997	4	Japan	25	39	17:8	CD 100%	EVF 80%; CVF 20%	
Walker	2002	1991–1995	2b	UK	19	68	8:11	DD 73.6%; CRC 15.7%; Trauma 5.2%; CD 5.2%	CVF 100%	
Menenakos	2003	1993–2002	2b	Greece	15	68.1	10:5	DD 100%	CVF 100%	
Najjar	2004	1992–2004	4	USA	12	63.9	12:0	DD 75%; CRC 16.7%; BC 8.3%	CVF 100%	
Kavanagh	2005	1990–2000	4	Ireland	25	63.5	13:12	DD 52%; CRC 16%; BC 4%; CD 12%; PR 12%; IA 4%	CVF 76.7%; RVF 13.3%; EVF 10%	
Laurent	2005	1992–2003	4	Belgium	16	60	10:6	DD 100%	CVF 100%	
Ferguson	2008	1993–2005	4	USA	74	54	48:26	DD 70.3%; CD 29.7%		
Melchior	2009	1982–2007	4	Germany	49	69.5	42:7	DD 100%	CVF 100%	
Lynn	2012	2003–2010	2b	USA	72	60	50:22	DD 65%; Malignancy 15%; CD 18%; Trauma 2%	CVF 85%; RVF 15%	
Niebling	2013	1998–2010	4	Netherlands	31	61.1	19:12	DD 100%	CVF 100%	
Maciel	2014	2009–2013	2b	USA	75	62.3	39:36	DD 100%	CVF 100%	
Salgado-Nesme	2016	2005–2011	2b	Mexico	24	56.5	22:2	DD 100%	CVF 100%	
Taxonera	2016	NR	2b	Spain	79	33	60:19	CD 100%	EVF 66.9%; CVF 31.9%	
Badic	2017	2000–2014	2b	France	28	68	20:8	DD 100%	CVF 100%	
El-Haddad	2018	2012–2017	4	Egypt	40	58	36:4	DD 100%	CVF 100%	
Nevo	2019	2007–2017	4	Israel	17	48 (median)	15:2	DD 59%; CD 41%	EVF 41%; CVF 59%	

*OCEBM* Oxford Center of Evidence Based Medicine, *CVF* colovesical fistula, *RVF* rectovesical fistula, *EVF* enterovesical fistula, *DD* Diverticular disease, *CD* Crohn disease, *CRC* colo-rectal cancer, *BC* bladder cancer, *PC* prostate cancer, *PR* post radiation, *IA* iatrogenic

The most frequent etiology of EVF/CVF described was a benign disease, with diverticular and Crohn’s diseases accounting for 60.2% (519 patients) and 27.5% (237) of all cases respectively. Malignant diseases (mainly colorectal cancer; but also, bladder and prostate cancer) accounted for 6.5% of all cases (56 patients). Among other causes, appendicitis, trauma, radiation and iatrogenic lesions comprised a total of 21 cases. Only one study (Munoz 1998) did not report adequate details regarding EVF or CVF etiology.

The diagnostic methods most frequently adopted were CT, barium enema, and cystoscopy, which were used on average in 62.5, 62.6, and 59% of cases respectively. Further details about diagnostic procedures are available in Additional file 1.

### Primary outcome

**Fig. 2:**
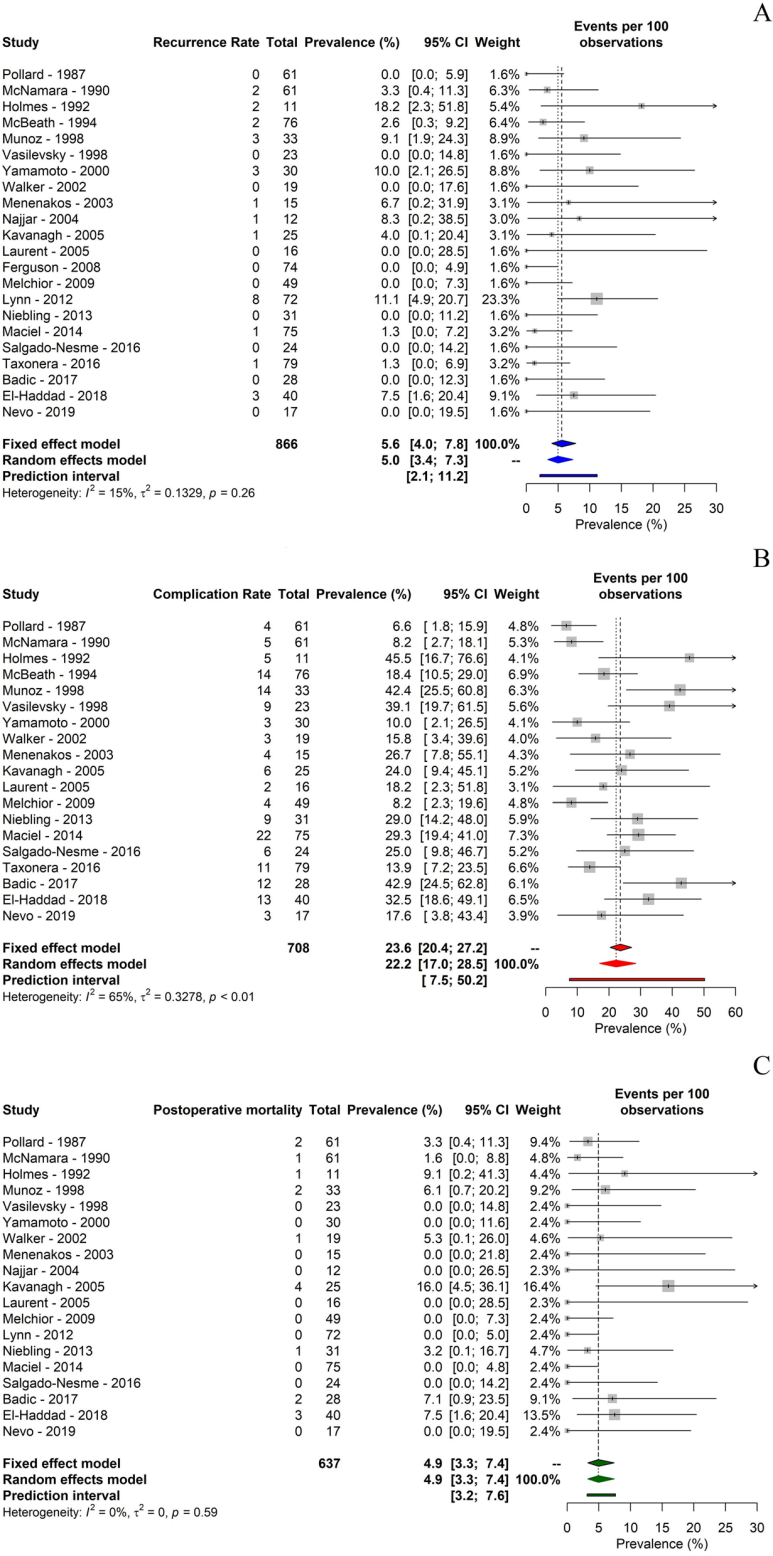
meta-analysis of proportions of **A** primary and **B**, **C** secondary outcomes

No outliers were identified. The meta-analysis of proportions pointed out a 5% recurrence rate of the fistula (95% CI 3.37–7.32). I^2^ statistics revealed the presence of low heterogeneity (I^2^ = 14.8%; p = 0.26) (Fig. 2A).

### Secondary outcome

Only 19 studies recorded information regarding postsurgical morbidity ranging from 6.6% up to 45.5%. The meta-analysis of proportions pointed out a 22.2% overall complication rate (95% CI 17.04–28.51). I^2^ statistics revealed the presence of moderate heterogeneity (I^2^ = 64.6%; p < 0.0001) (Fig. 2B).

Nineteen studies reported data regarding postoperative mortality: 10 of them reported no postoperative mortality, whereas in 9 studies a range from 1.6% to 16% was found. The meta-analysis of proportions pointed out a 4.9% postoperative mortality rate (95% CI 3.26–7.41). No heterogeneity was demonstrated (I^2^ = 0%; p = 0.58) (Fig. 2C).

Detailed data about recurrence, complications, and mortality rates are reported in Table 2.

**Table 2:** Recurrence, complications and postoperative mortality outcomes

Author	Median follow-up (months)	Recurrence rate (%)	Complication rate (%)	Complications (description)	Postoperative mortality (%)	
Pollard	195 (mean)	0	6.5	Cardiorespiratory complications 3.2; anastomotic leak 3.2%	3.2	
McNamara	106 (mean)	3.3	8.2	Urine leak 3.2%; enterocutaneous fistula 1.6%; wound infection 1.6%	1.6	
Holmes	13	18.2	45.5	Deep vein thrombosis 9.1%; chest infection 27.3%; pelvic abscess 9.1%	9.1	
McBeath	15.3 (mean)	2.8	18.6	Anastomotic leak 5.7%; small bowel obstruction 2.8%; ileus 1.4%; other 8.7%	NR	
Munoz	34	9.1	42	NR	6	
Vasilevsky	30.8	0	40	Urinary tract infection 9.5%; atelectasis 7.1%; prolonged ileus 4.8%; arrhythmias 4.8%; other 13.8%	0	
Yamamoto	159	10	10	Anastomotic leak 6.7%; intrabdominal abscess 3.3%	0	
Walker	35	0	15.7	Urinary leak 5.3%; wound infection 5.2%; ischemic colitis 5.2%	5.2	
Menenakos	61.7	5.5	27.7	Anastomotic leak 6.6%; gastroparesis 6.6%; abdominal wall hematoma 6.6%; minor hemorrhage through the drain 6.6%	0	
Najjar	2–108 (range)	8.3	NR	NR	0	
Kavanagh	6 (mean)	4	24	Acute renal failure 4%; acute pulmonary oedema 4%; atrial fibrillation 4%; incisional hernia 4%; wound infection 4%; anastomotic leak 4%	16	
Laurent	64 (mean)	0	18.2	Pulmonary infection 9.1%; postoperative hemorrhage 9.1%	0	
Ferguson	6.5	0	NR	NR	NR	
Melchior	68	0	8.2	Pneumonia 6.1%; wound infection 2%	0	
Lynn	31	11.1	NR	NR	0	
Niebling	129	0	29	Wound infection 12.9%; urinary tract infection 6.5%; abscess formation 6.5%; anastomotic leak 3.1%	3.2	
Maciel	8.8 (mean)	1.3	29.3	Colocutaneous fistula 2.6%;	0	
Salgado-Nesme	18.6 (mean)	0			0	
Taxonera	101	1.3	13.9	Abdominal abscesses 6.3%; anastomotic leaks 2.5%; intestinal obstruction 1.3%; hemorrhage 3.8	NR	
Badic	12	0	43	NR	7	
El-Haddad	21	7.5	32.5	Anastomotic leak 7.5%; parastomal hernia 5%; colostomy retraction 2.5%; ileus 7.5%	7.5	
Nevo	49	0	18	Surgical site infection 6%; intra-abdominal abscess 6%; persistent postoperative fever 6%	0	

*NR* not reported

### Metaregression analysis

Metaregression analysis highlighted a trend towards a decrease of fistula recurrence over time with a 1% drop per year (Risk Ratio 0.989; 95% CI 0.94–1.03), although this result was not significant (p = 0.65). Bubble plot of metaregression is displayed in Additional file 1.

### Assessment of publication bias

**Fig. 3:**
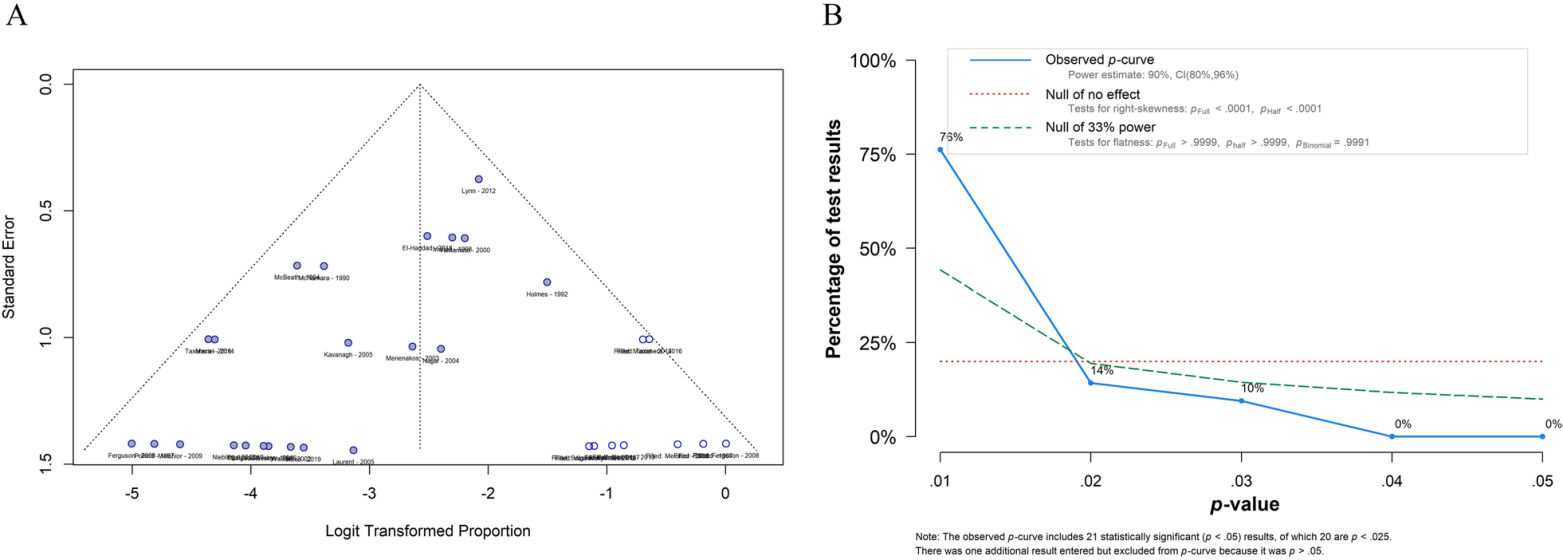
**A** Funnel plot of publication bias; **B** P-curve analysis plot

Egger’s test of recurrence rate meta-analysis of proportions pointed out significant asymmetry (p = 0.00006). P-curve estimates of 21 studies showed a 90% power of analysis (95% CI 80–96%) with a significant right skewness of the curve (p < 0.001), underlining a “true” effect size behind our findings. Funnel plot of publication bias and P-curve analysis plot is shown in Fig. 3.

Further results are reported in Additional file 1.

### Surgical management

In most of the studies, a one-stage laparotomic approach was performed. Eighteen studies reported the use of open surgery for a total of 637 patients. The laparoscopic approach was reported in only 7 studies for a total of 121 patients, with an average conversion rate to open surgery of 23%. The study published by Maciel et al. was the only reporting robotic approach for the treatment of 20 CVF patients; in their series, no need for conversion to open surgery was recorded [37].

Primary resection and anastomosis (one-stage procedure) was performed in 641 patients (75.5%). A multi-stage strategy with curative intent, comprehensive of resection and primary anastomosis with colostomy and/or Hartmann procedure (two-stage procedure) with later closure of the stoma (three-stage approach), was carried out in 132 patients (15.5%). Interventions with palliative intent (i.e., definitive ileostomy or colostomy) were performed in 53 patients (6.2%) mainly due to locally advanced/metastatic neoplastic disease or poor general conditions. Only one study, with a total of 23 patients, did not report details regarding the surgical approach [29].

Seventeen studies described the surgical procedures performed on the bladder, providing data on 662 out of 849 patients. In 215 patients (32.4%), minimal bladder surgery (i.e., simple blunt dissection, fistula division, omental interposition, debridement, curettage) was sufficient. On the other hand, direct repair (with suture or with mechanical stapler) was performed on 344 patients (51.9%). Partial or radical cystectomy was indicated in a small proportion of patients, respectively 92 (13.8%) and 19 (2.8%) cases.

All of the studies reported the need for extensive vesical catheterization after surgery. Eleven of them reported a mean catheterization time of 9.2 days, ranging from 7 to 14 days. Further details are reported in Table 3.

**Table 3:** Surgical management details

Author	Surgical approach	Conversion rate (%)	Intestinal surgery				Bladder surgery	Mean catheterization time (days)	Mean length of stay (days)
			Single stage (%)	Two-stage (%)	Three-stage (%)	HP/definitive ileostomy or colostomy/other palliative interventions			
Pollard	Open 100%		55.7	22.9	11.5	9.9 (HP: 4.95)	Simple dissection (% NR); partial cystectomy 3.2%; direct repair 1.6%		
McNamara	Open 100%		75.4	24.6			Primary closure 34.4.%; excision and closure 16.4%; left open 49.2%	10	14
Holmes	Open 100%		63.6	36.4			Partial cystectomy 54.6%; cystectomy and ileal loop 18.2%; cystectomy and urostomies 18.2%; cystectomy and uretric reimplantation 9.1%		
McBeath	Open 100%		65.7	25.7		8.6	NR		
Munoz	Open 100%		27.3	12.1		60.6	NR		
Vasilevsky	Open 100%						Direct repair 100%	7	
Yamamoto	Open 100%		100				Direct repair 100%	14	18
Walker	Open 100%		78.9	5.2		HP: 15.7	No bladder repair 94.8%; partial cystectomy 5.2%	10	
Menenakos	Laparoscopic 100%	5.5	100				Simple dissection (% NR); suture with device 40%; excision and closure 13.3.%	9	10
Najjar	Open 100%		83.4	8.3	8.3		NR		
Kavanagh	Open 96%; Laparoscopic 4%		48	36		16	Excision of a cuff of bladder 100%		29.4
Laurent	Laparoscopic 100%	18.7	100				Direct repair 63.4%		5.7
Ferguson	Open 100%		100				Direct repair 25.7%; omentoplasty 6.8%	7	
Melchior	Open 100%		100				Direct repair 100%	7	\
Lynn	NS	NS	61	39			Simple repair 69.4%; advanced repair 30.6%		
Niebling	Open 100%		93.5			6.5	Direct repair 100%	7	
Maciel	Laparoscopic 73.3%; Robotic 26.7%	14.5	96	4			NR		4.1
Salgado-Nesme	Open 50%; Laparoscopic 50%	25	83.4			16.6	NR	10	9 (open) / 15 (laparoscopic)
Taxonera	Open 85%; Laparoscopic 15%	33.3	88.7	6.3		5	Direct repair 100%		14 (open) / 7 (laparoscopic)
Badic	Open 50%; Laparoscopic 50%	43	100				Direct repair 64%	10	12.9 (open) / 13.1 (laparoscopic)
El-Haddad	Open 100%		40	40	12.5	7.5	Minimal bladder surgery 45%; partial cystectomy 52.5%; ureter resection/re-anastomosis 7.5%	10	
Nevo	Laparoscopic 100%	0	100				Simple dissection 30%; direct repair 70%	2–7	7 (median)

*NR* not reported, *HP* Hartmann procedure

## Discussion

Managing entero-colovesical fistulas can be challenging. Much of our knowledge results from the opinions of few experts in the field, emerging from large case series rather than from trial settings. Consequently, there is a wide variation in the definitions of fistula location and complexity with little standardization of treatment protocols and outcome measures [42]. Since the closure rate of the fistula remains quite low after conservative treatment and due to the remarkable risk of septic complications following conservative treatment, all patients able to tolerate surgery, should be deemed surgical candidates. The lack of consensus on the *if, when, and how* to repair a fistula represents a crucial issue for surgeons and the development of standardized and well-structured protocols of treatment are unmet key needs, urgently required to ensure patient safety. An important consideration regards the choice of diverting stoma. Seventeen studies reported information regarding the type of ostomy. Colostomy was performed in 69 cases, whereas ileostomy in 19 cases. The proportion of the former was more than triple compared to the latter. This finding may be explained considering the greater proportion of diverticular disease-related fistulas. On the other hand, ileostomy was performed mainly as palliative treatment in the presence of frozen pelvis caused by radiation therapy or advanced cancer.

Kavanagh reported the highest mortality (four patients over 25–16%). All patients who died suffered from diverticular disease. A possible explanation for this finding would be the advanced age (80 years) of this subset of patients. Albeit not significant, metaregression analysis pointed out an inversely proportional correlation between recurrence rate and years of publication. This may reflect a progressive improvement in surgical techniques over time.

### Surgical management of the enteric tract

The choice of the best surgical strategy relies on the underlying etiology of the fistula. In any case, the goal is the resection of the intestine/bowel segment involved in the fistula and the closure of the bladder. Surgical treatment options can be summarized as single-stage and multi-stages procedures. Due to the low mortality and similar postoperative morbidity rates compared to staged procedures, primary resection, and anastomosis (single-stage repair) should be considered the intervention of choice whenever feasible [5, 27]. A single-stage procedure was possible in more than 90% of the cases in 8 out of 21 studies of the present review. Among others, El-Haddad and Lynn reported the highest rates of multi-stages procedures, 52.5% and 39% respectively [9, 35]. Although no differences between single- and multi-stage procedures in terms of fistula recurrence were observed, a two-stage repair (primary anastomosis with colostomy/ileostomy or Hartmann procedure) was considered the optimal surgical strategy for patients with an inflammatory colon mass. Primary anastomosis with ileostomy should be also be thoroughly considered for CD patients, especially those presenting with ileo-vesical fistulas. This decision should also encompass risk factors associated with higher morbidity (perioperative steroid use, smoking, malnutrition), as well as the degree of inflammation/quality of surrounding tissues. Three-stages repair, characterized by defunctioning ostomy followed by resection-anastomosis and later closure of the stoma, was preferred in patients with pelvic abscess and poor performance status. A definitive diverting ileostomy or colostomy is mostly performed with palliative intent, it is rarely associated with fistula healing, and carries a high risk of persistent/recurrent urinary sepsis. Nevertheless, it can represent a valid strategy to relieve patients’ symptoms. Other candidates for defunctioning stoma are represented radiation-induced fistula patients; due to the high risk of recurrence and intraoperative technical difficulties encountered in severe cases, this kind of surgery can sometimes represent the only treatment option [1].

Although open surgery remains the preferred approach to manage EVFs/CVFs, minimally invasive procedures have progressively been adopted in surgical practice. Laparoscopic surgery has been reported to have similar fistula recurrence rates and postoperative complications. In the present review, 7 studies for a total of 121 patients reported data of minimally invasive procedures. Most of them claim that laparoscopic surgery has been demonstrated safe and feasible in experienced hands, although the conversion rate remains remarkable [37]. In our analysis conversion to open surgery ranged from 5.5% [32] to 33.3% [39] and an average complication incidence of 25.5% was computed.

### Surgical management of the bladder

A great debate surrounding the optimum timing of repair, immediate or delayed, still exists. While the exact definition of what constitutes an “immediate” repair varies between studies, most authors consider less than 6 weeks appropriate. Intuitively, repair should be performed following a period of catheterization to allow the inflammation to settle and necrotic material to slough off whilst providing the opportunity for spontaneous closure[42]. A surgical repair of the bladder should not be routinely performed; indeed, according to Carpenter et al. in case of small fistulas, bladder catheterization alone might be sufficient [43]. In this regard, Walker et al. highlighted how the resection of the fistula with primary bowel anastomosis and bladder drainage alone resulted in no recurrences and low morbidity [31]. Similarly, Ferguson et al., in their series of 74 patients with EVFs of benign etiology, observed that indwelling Foley catheter placement alone is sufficient for bladder healing [34].

Available evidence suggests that, in case of large fistulas/overt defects into the bladder, a direct repair of the bladder wall with suture (single or double layer) shows favorable outcomes in terms of fistula recurrences and complication rates. In this case, simple suture, fistula curettage followed by suture, or omentum flap between bowel anastomosis and bladder represent valuable options [34]. Aggressive approaches such as partial or radical cystectomy as first-line treatment for EVF/CVFs should be considered only in case of infiltrating bladder cancer [44]. Indeed, in our analysis, only 10% and 3% of patients underwent partial and radical cystectomy respectively. The risk of fistula recurrence in bladder repair has been demonstrated to be four times greater in bladder wall resection as compared with primary repair [35]. The best timing for catheter removal is still debated, ranging from 7 to 14 days and mainly depending on bladder repair complexity and the primary cause of fistula occurrence.

### Strengths and limitations

Other valuable systematic reviews previously published in the literature provided a comprehensive description of the diagnostic and therapeutic management of patients suffering from EVFs/CVFs. To the best of our knowledge, the present study is the first to focus specifically on surgical treatment of these patients and assessing recurrence, complications, and mortality rates through meta-analytic computations. Nevertheless, it is worth underlining some limitations. The quality of evidence was quite low, with all studies being retrospective. The diagnostic evaluation and follow-up of patients with enterovesical fistulas were heterogeneous across the included studies and only a minority of reports described the specific diagnosis, treatment, and scheduled imaging follow-up. Moreover, indications for surgical repair were either not reported or not standardized in most series and relied mainly on the surgeon’s judgment. Finally, since most of the studies did not report separate outcome data for different surgical procedures nor different etiologies, the possibility to develop pair-wise comparisons as well as to explore the effect of clinically relevant variables on fistula recurrence was precluded.

## Conclusion

In conclusion, our analysis has demonstrated that, although burdened by a non-negligible rate of complications, surgical repair of entero-colovesical fistula leads to favorable results in terms of primary healing. Our review offers opportunities for significant further research in this field. Future prospective studies with granular datasets should evaluate the benefits and harms of surgical treatment using standardized and well-scheduled diagnostic examinations in patients with suspicion of enterovesical fistulas.

## Supplementary Information

**Additional file 1**. Supplemetary materials including diagnostic methods, results of sensitivity analysis, funnel plots and P-curve analysis for complications and postoperative mortality, bubble plot of metaregression, word combination syntax.

## Data Availability

The data and code that support the findings of this study are available from the corresponding author, upon reasonable request.

## Abbreviations

EVF: Enterovesical fistula
CVF: Colovesical fistula
RVF: Rectovesical fistula
CT: Computed tomography
MR: Magnetic resonance
CD: Crohn’s disease
DD: Diverticular disease
CRC: Colorectal cancer
BC: Bladder cancer
PC: Prostate cancer
PR: Post radiation
IA: Iatrogenic
FUP: Follow-up
OCEBM: Oxford Center of Evidence Based Medicine
