# Supplementary material for: Indications and outcomes of enterovesical and colovesical fistulas: systematic review of the literature and meta-analysis of prevalence
Source: BMC Surg. 2021 May 27;21:265. doi: 10.1186/s12893-021-01272-6 (PMC8157688; doi:10.1186/s12893-021-01272-6)
Supplement: Supplementary file 1 — Additional file 1. Supplemetary materials including diagnostic methods, results of sensitivity analysis, funnel plots and P-curve analysis for complications and postoperative mortality, bubble plot of metaregression, word combination syntax. [file 12893_2021_1272_MOESM1_ESM.docx]

**Indications and outcomes of enterovesical and colovesical fistulas: systematic review of the literature and meta-analysis of prevalence**

**Supplementary Materials**

**Index**

| 1. Table 4: diagnostic methods | 2 |
| --- | --- |
| 1. Sensitivity analysis - Baujat plot | 3 |
| 1. Funnel plot and p-curve analysis for complications | 4 |
| 1. Funnel plot and p-curve analysis for mortality | 5 |
| 1. Bubble plot of metaregression: years of publication | 6 |
| 1. Word combination syntax | 7 |

1. **Table 4: diagnostic methods**

| **Author** | **Year of publication** | **Diagnostic method** | | | | | |
| --- | --- | --- | --- | --- | --- | --- | --- |
|  |  | **CT (%)** | **Endoscopy (%)** | **Cystoscopy (%)** | **Cystography (%)** | **Barium enema (%)** | **Other** |
| Pollard | 1987 |  | 62.1 | 60.6 | 7.5 | 80.3 | IV pyelography: 66.6% |
| McNamara | 1990 |  | 15.8 | 63.5 | 25.4 | 60.1 | IV pyelography: 69.8%; small-bowel series 63.5% |
| Holmes | 1992 | - | - | - | - | - |  |
| McBeath | 1994 | 42.1 | 35.5 | 55.2 | 35.5 | 76.3 | IV pyelography: 38.1%; oral charcoal 5.2% |
| Munoz | 1998 | 54.5 | 78.8 | 96.9 | 60.6 | 30.3 | IV pyelography: 39.4% |
| Vasilevsky | 1998 |  |  | 100 | 100 | 100 |  |
| Yamamoto | 2000 | - | - | - | - | - |  |
| Walker | 2002 | 36.8 | 10.5 | 21 |  | 84.2 |  |
| Menenakos | 2003 | - | - | - | - | - |  |
| Najjar | 2004 | 83 | 25 | 50 | 75 | 83 | IV pyelography: 58% |
| Kavanagh | 2005 | 50 | 36.6 | 53.3 | 3.3 | 33.3 | Oral charcoal 16.6% |
| Laurent | 2005 | 9.1 |  | 45.4 |  | 90.9 |  |
| Ferguson | 2008 | 100 |  | 100 |  |  |  |
| Melchior | 2009 | 83.7 | 95.9 | 100 | 61.2 | 85.7 | Poppy seed test: 75.5%; MR 10.2% |
| Lynn | 2012 | - | - | - | - | - |  |
| Niebling | 2013 | 90.3 | 83.9 | 54.8 |  | 74.2 |  |
| Maciel | 2014 | - | - | - | - | - |  |
| Salgado-Nesme | 2016 | - | - | - | - | - |  |
| Taxonera | 2016 | 53.6 | 5.1 | 12.4 | 10.3 | 3 | MRI 59.8 |
| Badic | 2017 | 46.4 | 61 | 18 |  |  |  |
| El-Haddad | 2018 | 100 | 90 | 55 | 17.5 | 12.5 |  |
| Nevo | 2019 | - | - | - | - | - |  |

1. **Sensitivity analysis – Baujat plot**


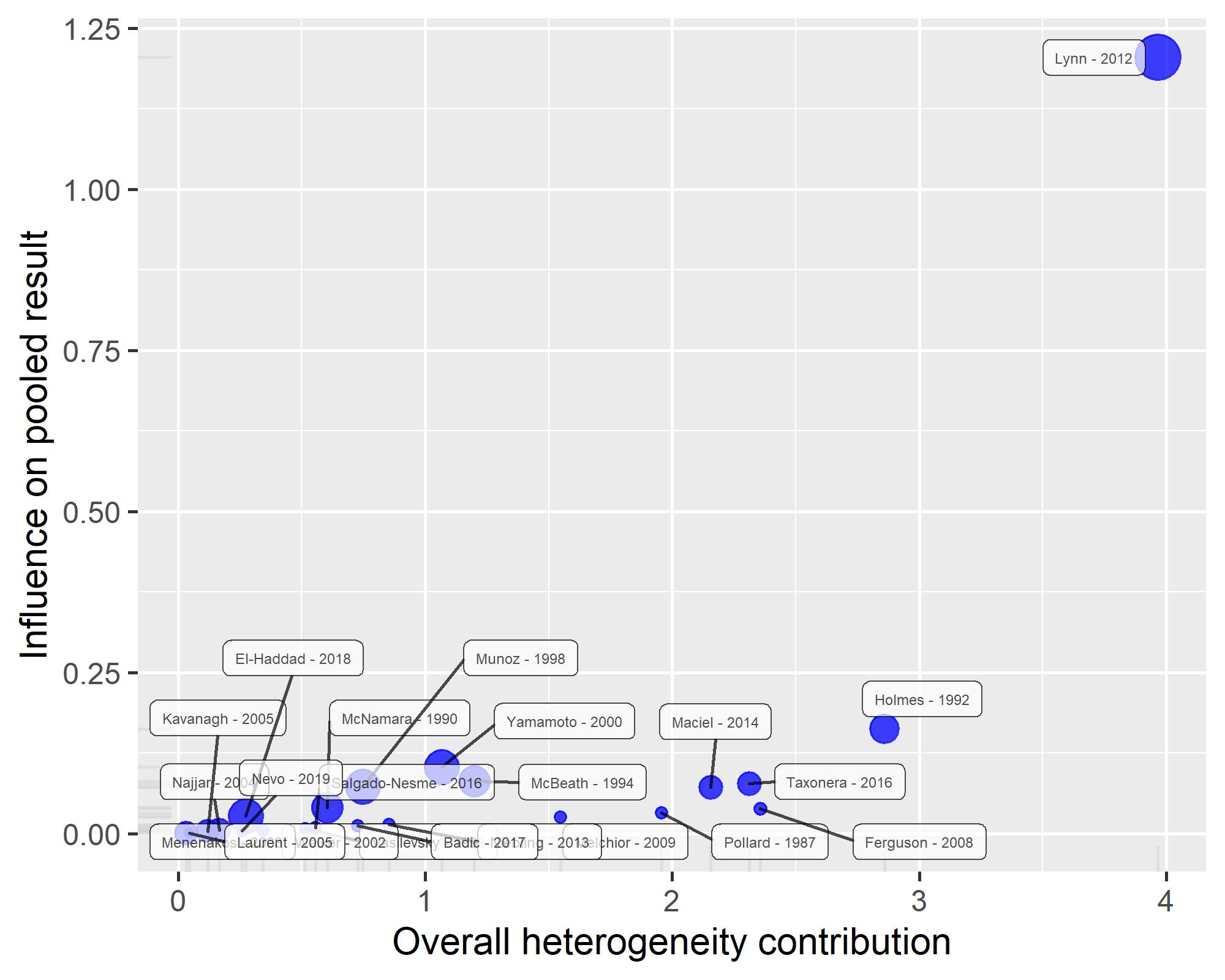


1. **Funnel plot and p-curve analysis for complications**

**
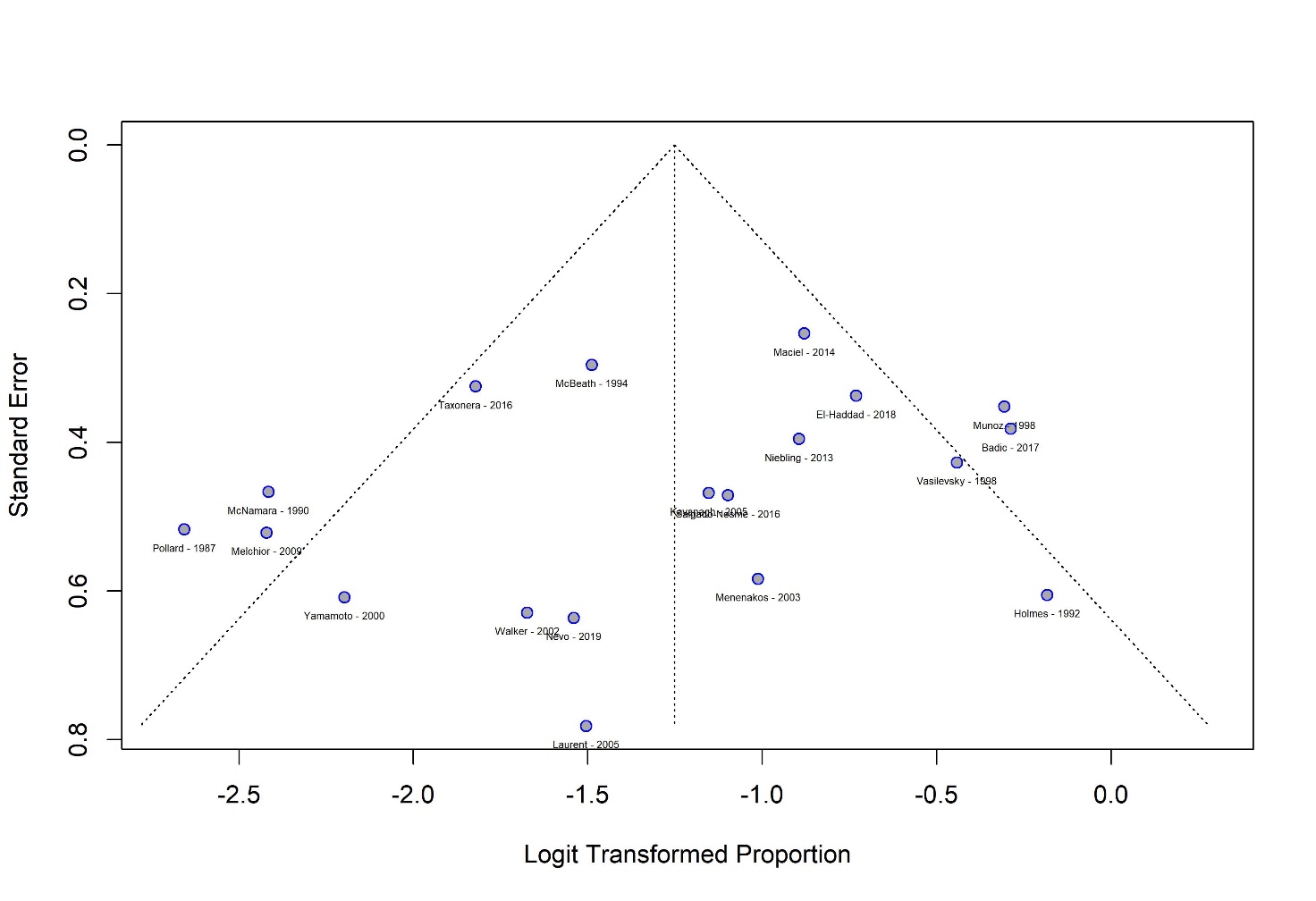
**

**
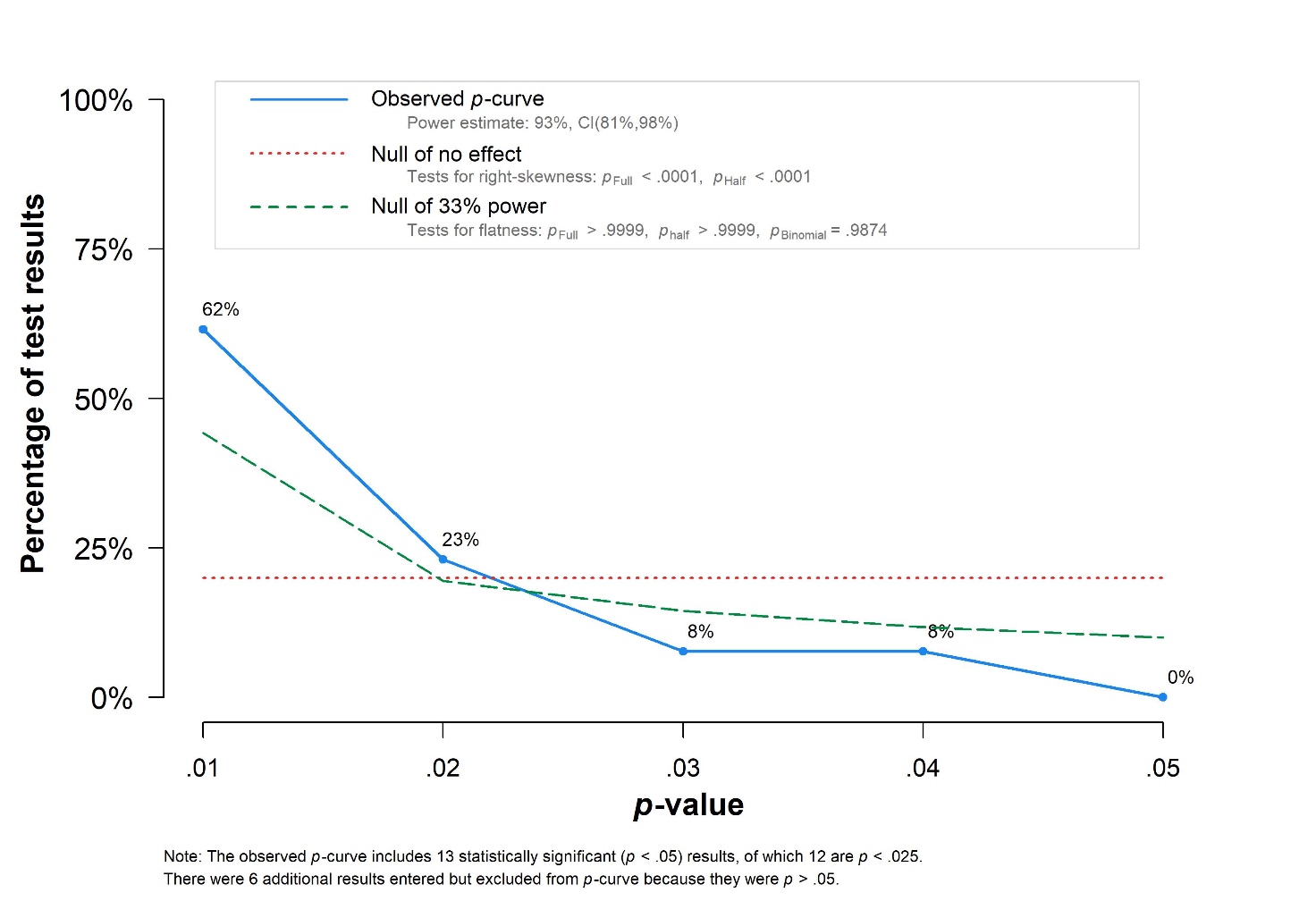
**

1. **Funnel plot and p-curve analysis for mortality**

**
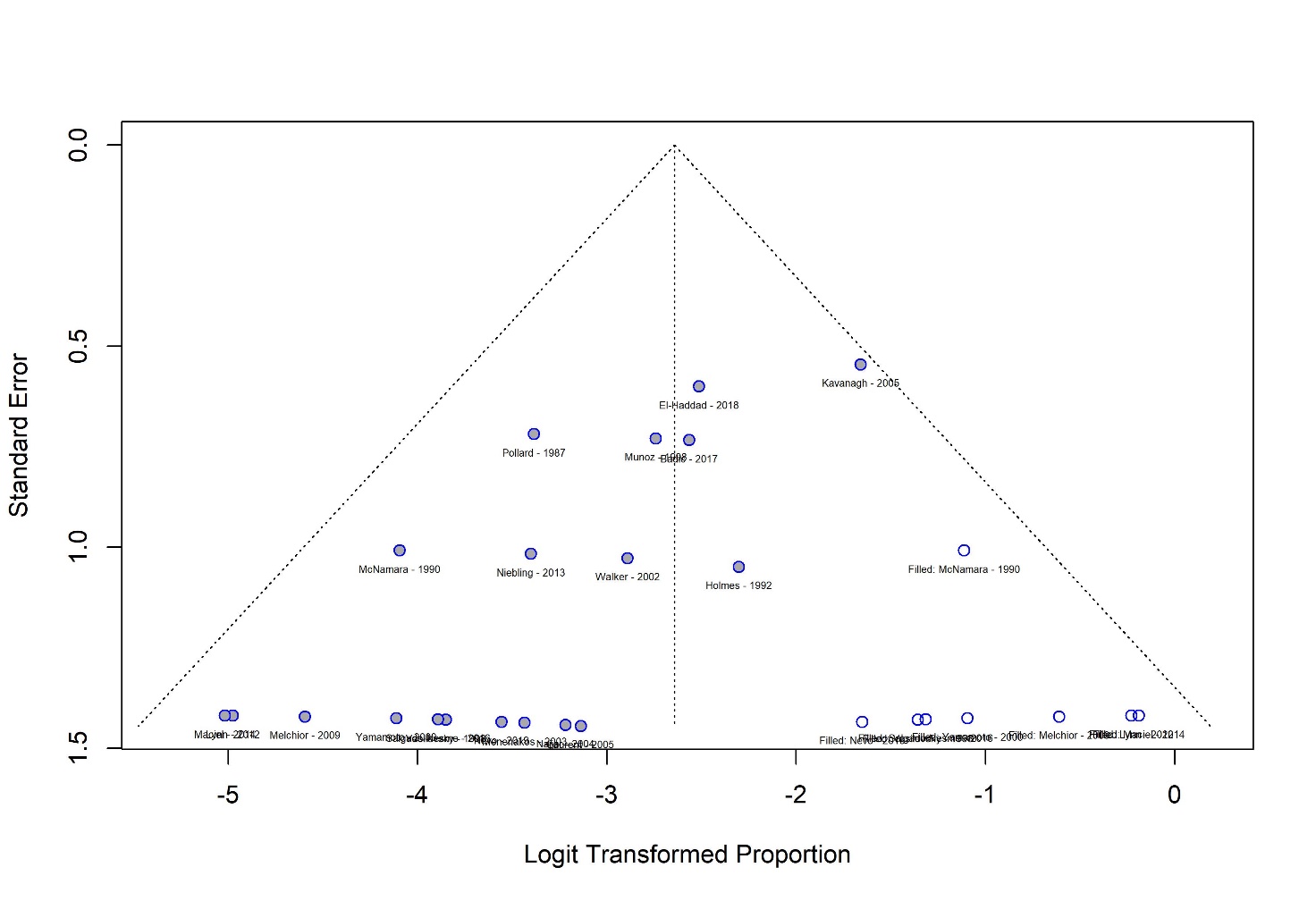
**

**
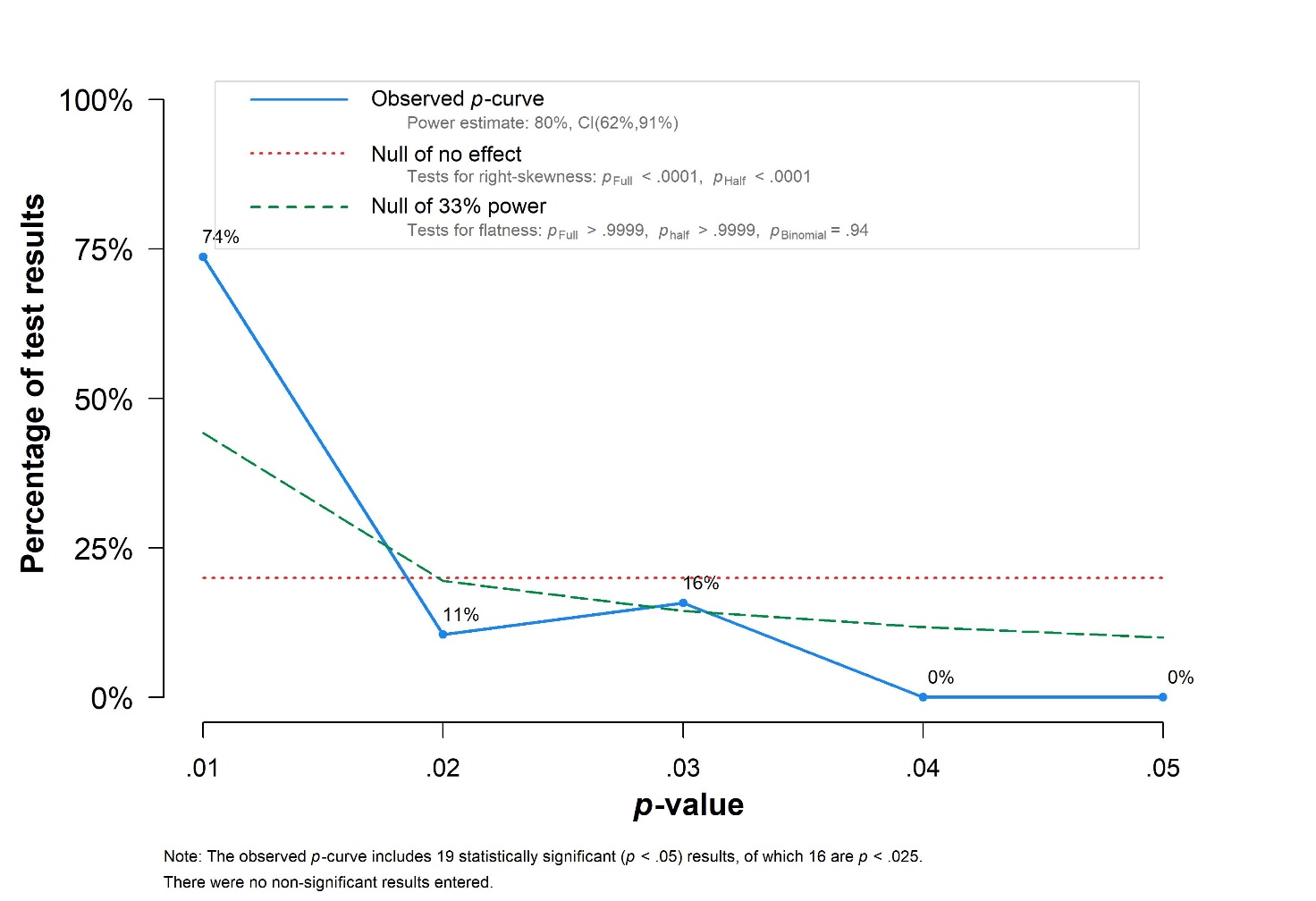
**

1. **Bubble plot of metaregression: years of publication**


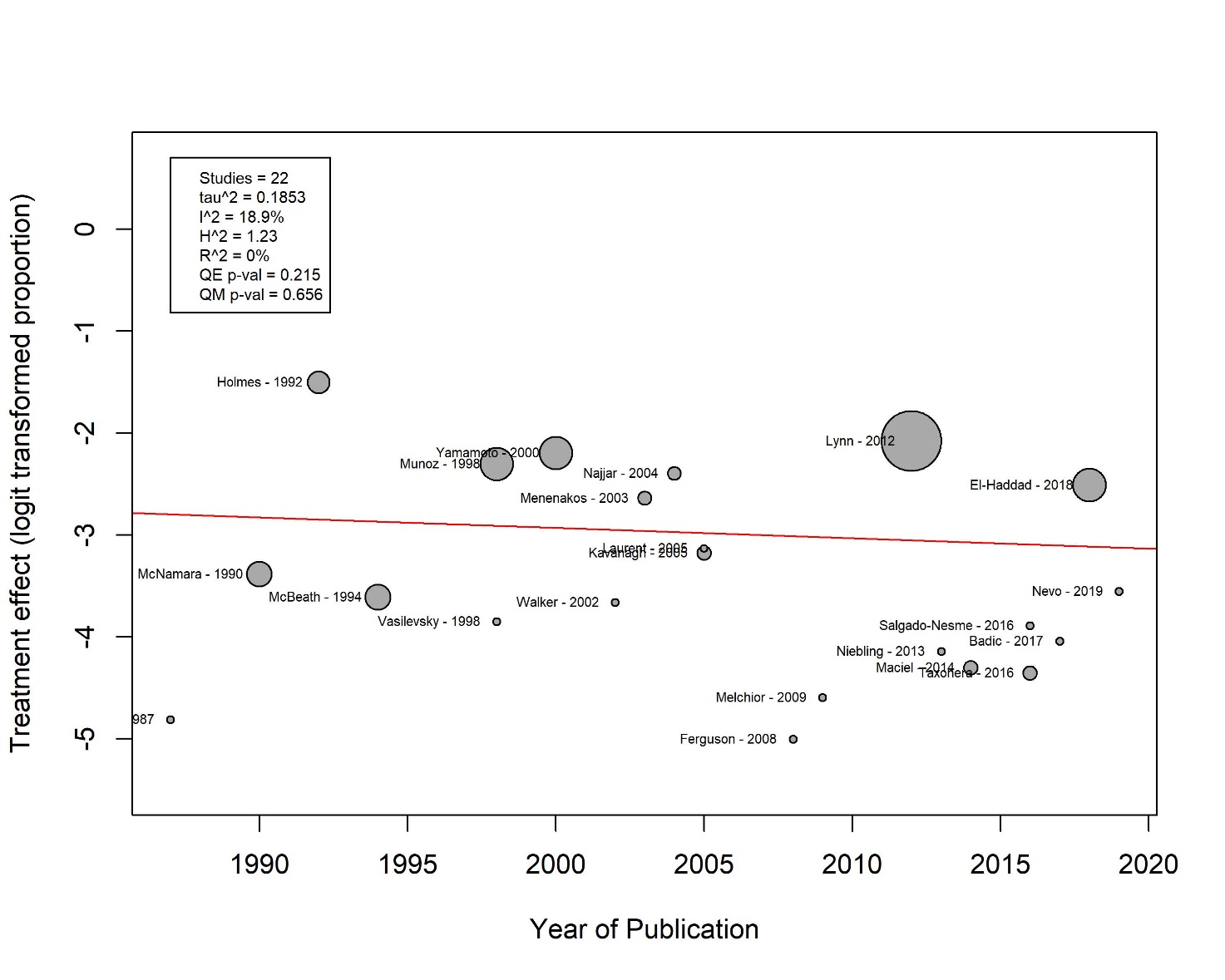


1. **Word combination syntax**

Keywords used for database search: “colovesical fistula”, “enterovesical fistula”, “pelvic fistula”, “surgery”, “surgical treatment”, “surgical management”, “primary healing”, and “outcome”. The keywords “enterocutaneous”, “enteroatmospheric”, “anal fistula” and “wound healing” were set to be excluded from the search.

Search query: ((colovesical fistula) OR (enterovesical fistula) OR (pelvic fistula)) NOT (enterocutaneous) NOT (enteroatmospheric) NOT (anal fistula) AND ((surgery) OR (surgical treatment) OR (surgical management)) AND ((primary healing) OR (outcome)) NOT (wound healing)
